# Nanostructure-enhanced infrared spectroscopy

**DOI:** 10.1515/nanoph-2021-0661

**Published:** 2021-12-03

**Authors:** Takuo Tanaka, Taka-aki Yano, Ryo Kato

**Affiliations:** Metamaterials Laboratory, RIKEN Cluster for Pioneering Research, 2-1 Hirosawa, Wako , Saitama, 351-0198, Japan; Innovative Photon Manipulation Research Team, RIKEN Center for Advanced Photonics, 2-1 Hirosawa, Wako, Saitama, 351-0198, Japan; Institute of Post-LED Photonics, Tokushima University, 2-1 Minami-Josanjima , Tokushima, 770-8560, Japan

**Keywords:** infrared spectroscopy, nano-imaging, nanostructure, metamaterials, SEIRA, surface plasmons

## Abstract

While infrared spectroscopy is a powerful technique that provides molecular information such as chemical constituents and chemical structures of analytes, it suffers from low absorption cross-section resulting in low sensitivity and poor signal-to-noise or signal-to-background ratios. Surface-enhanced infrared absorption (SEIRA) spectroscopy, which is supported by nanometer scale structures, is a promising technology to overcome these problems in conventional infrared (IR) spectroscopy and enhances IR signals using the field enhancement properties of surface plasmon resonance. Recently resonant SEIRA technique was proposed, and signal enhancement factor was significantly improved. In this review, we present an overview of the recent progresses on resonant SEIRA technologies including nanoantenna- and metamaterial-based SEIRA, and also SEIRA techniques with nanoimaging capabilities.

## 1 Introduction

Spectroscopy is a powerful optical and photonic technique. It provides large amounts of information. We can easily distinguish a fresh apple on a table from a rotten or underripe one through their skin colors, even though it is limited to the visible frequency region. Since our naked eyes cannot perform spectroscopy, our discrimination of fresh apples does not rely on the spectrum of the apples’ skin. If we measure the spectrum of the color by spectroscopy, we can obtain much more important material information. Particularly, because the frequency of infrared (IR) light matches that of vibrational and rotational motion of molecules, the IR spectrum contains information about the chemical structures and constituents of materials. Therefore, IR spectroscopy is an important technique and is widely used in various fields, including chemistry, biology, pharmacy, environment, material science, and so on. It also has applications in areas of safety, food, and the identification of substances.

However, because the absorption cross-section of molecules in the IR spectral region is relatively low, and all heat objects surrounding us always radiate IR light, IR spectroscopy suffers from the low-signal intensity and highly unwanted backgrounds and noise, resulting in poor signal-to-noise (S/N) and signal-to-background (S/B) ratios. Therefore, it is difficult to measure a small number of analyte molecules. To solve this problem, several efforts have been made, and many signal-enhancing techniques, such as attenuated total reflectance (ATR) and grazing incidence reflectance, have been developed. For example, reflection absorption spectroscopy (RAS) is a grazing incidence reflectance technique. It uses metal plates/films, and the incident light is introduced to the metal surface at a large incident angle with a p-polarized orientation. Owing to the interferential interaction of incident and reflected light, the amplitude of the electric wave near the metal surface doubles, resulting in a four-times enhancement of the intensity of the light. RAS uses intensified electric field and increases the molecular signals from analyte molecules adherent on the surface of a metal.

To improve the weak signals from molecules, several methods have been developed. Surface-enhanced IR absorption (SEIRA) enhances IR signals using the field enhancement properties of surface plasmon resonance at tiny metal particles/structures. Experimental realization of SEIRA was reported in 1980 by Hartstein et al. [1]. Using a 6 nm-thick silver (Ag) film, which was randomly arranged Ag nanoislands, deposited on the surface of a silicon ATR prism, an enhancement factor of 20 was achieved. Both theoretical and experimental studies on SEIRA have been intensively conducted by Osawa [2]. Resonant SEIRA, which leverages resonant interactions between broadband plasmons of metal structure and narrowband vibrational signal of molecules, was first demonstrated by Neubrech et al. in 2008 using a single gold nanowire [3]. Using resonant SEIRA, its signal enhancement factor was significantly improved by five orders of magnitude. Since then, SEIRA has attracted much attention, and numerous studies have been reported.

Based on this, herein, we summarize the recent progress on nanostructure-enhanced IR spectroscopy, focusing on their device structures. In Section 2, we review SEIRA techniques leveraging plasmonic nanostructures, such as metal nanoparticles and an array of them. In Section 3, we discuss metamaterial-based SEIRA techniques. Since it is difficult to separate these two topics, we categorize SEIRA supported by simple nanoantenna structures or having broad and weak plasmonic absorption bands as nanoantenna-based SEIRA, and that using rather complicated or artificial structures and strong resonant absorption close to unity is categorized as metamaterial-based SEIRA. In the last section, we review SEIRA-based techniques that have imaging capabilities with nanoscale high spatial resolutions.

## 2 Nanoantenna-based SEIRA

### 2.1 Plasmonic nanostructures

Local enhancement of electromagnetic fields is the dominant contribution to SEIRA. Plasmonic nanostructures are extremely promising field enhancers in the mid-infrared (MIR) regime, which effectively confine incoming infrared electromagnetic fields in their vicinity. Several MIR-resonant metallic nanostructures exhibit plasmon resonances at the same frequency as molecular vibration. Gold island thin films with randomly-formed plasmonic hot spots were mostly employed in early studies. Over the last two decades, remarkable advances in nanofabrication techniques have ensured more organized nanostructures with well-defined geometries (shape, size, and arrangement), which enable precise and fine regulation of electromagnetic resonances across specific vibrational frequency ranges in the MIR regime. Various well-shaped SEIRA-active structures, such as nanonails [4], split rings [5], nanoslits [6], and nanopedestals [7], have been reported. Among them, rod-like metallic nanoantennas, also known as nanowires or nanobars, have been commonly used as SEIRA-active nanostructures [3]. Metallic nanorods rely on longitudinal dipole resonances, resulting in strong field enhancement at both ends of the nanorods. The resonant wavelength of nanorods is tailored by simply controlling their aspect ratios. Gold nanorods require relatively high aspect ratios of 20–40 for MIR resonance and 2–4 for visible resonance. Semenyshyn et al. detected SEIRA from monolayered biomolecules using a single gold nanoantenna, paving the way to achieve ultimate detection limits of a few or even single-molecule levels [8]. Wei et al. designed and fabricated crooked nanorod antennas with suppressed radiative losses, achieving a 25-times enhancement of SEIRA compared to that of the commonly-utilized straight nanorod antennas [9].

**Figure 1:**
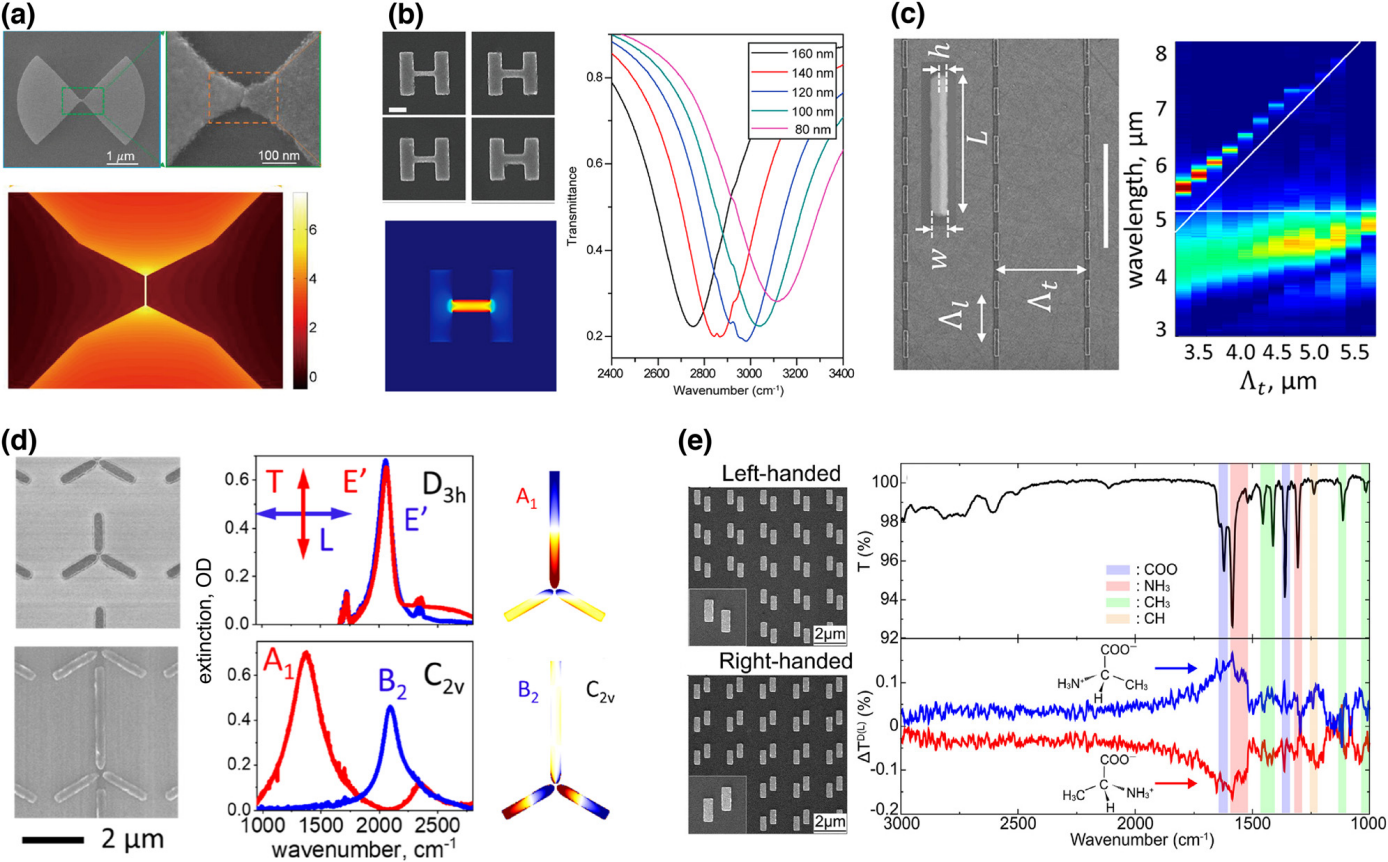
Plasmonic nanoantenna-based SEIRA structures. (a) Bowtie-shaped Au antenna: SEM images of an antenna with a sub-3 nm gap and calculated electric field distribution in the vicinity of gap at a resonant frequency. (b) Charge transfer plasmon (CTP) antenna: SEM images of the gold antennas with various bridge widths and width-dependent SEIRA spectra. (c) Periodic arrays of half-wavelength infrared antennas: their SEM images and optical extinction spectra of the arrays with various transverse periods. (d) Symmetry-broken trimer infrared gold antenna: SEM images and experimental polarization-selective extinction spectra. (e) Chiral gold antenna of staggered nano-rods: SEM images of left- and right-handed antennas and their application to vibrational circular dichroism spectroscopy. [(a) Reproduced with permission from reference [10]. Copyright © 2017, American Chemical Society; (b) reproduced with permission from reference [13]. Copyright © 2019, American Chemical Society; reproduced with permission from reference [16]. (c) Copyright © 2021, American Chemical Society; (d) reproduced with permission from reference [19]. Copyright © 2019, American Chemical Society; (e) reproduced with permission from reference [30]. Copyright © 2020, American Institute of Physics.]

Besides single nanorods, a nanogap antenna, consisting of two nanorods with nanoscale separation distance, provides much higher field enhancement near a gap, further lowering the detection limit of SEIRA. Inspired by such a pronounced field enhancement at a gap, Dong et al. used plasmonic bowtie nanoantennas [Figure 1(a)] with a sub-3 nm gap designed to provide a theoretical SEIRA enhancement factor of 10^7^ [10]. Optimized bowtie nanoantennas with an ultrasmall nanogap allowed for the detection of as few as 500 molecules of 4-nitrothipphenol. Chen et al. created not lateral but vertical nanogaps between elevated gold nanorods and complementary nanoslits on a substrate, which simultaneously excites dual MIR-resonant modes (polarization parallel and perpendicular to the nanorods) for MIR sensing [11].

Implementation of charge transfer plasmon (CTP) is a prospective approach for realizing smaller MIR-active plasmonic nanoantennas, enabling ultracompact SEIRA-active chips. CTP is induced by a nanoantenna dimer with a conductive junction that initiates direct charge transfer from one nanoantenna to the other across a conductive gap [12]. CTP resonance appears at wavelengths longer than the bonding dipole plasmon of the dimer. Wang et al. designed an MIR-active CTP antenna consisting of two gold rectangular antennas connected by a rectangular bridge [Figure 1(b)] [13]. The CTP resonance is linearly proportional to the square root of the bridge width; hence, it covers wide MIR frequencies (2500–3200 cm^−1^) by controlling the width. The array of CTP antennas further improved the detection sensitivity of SEIRA, enabling the detection of monolayers of 1-hexadecanethiol molecules.

Periodically arranged metallic nanostructures exhibit surface lattice plasmon (SLP) resonance at wavelengths close to the Rayleigh anomaly of the arrays. The SLP resonant modes enable the incident light to induce stronger near-field excitation [14]. Adato et al., for the first time, implemented SLP resonance for SEIRA spectroscopy [15]. They used periodic gold nanorod arrays to detect proteins and achieved a large enhancement factor of SEIRA (up to 10^5^). In addition, SLP resonance provides extremely narrow lines owing to the destructive interference in the array, leading to strong coupling between the narrow resonance and molecular vibrations. Cohn et al. recently demonstrated a significant narrowing of lattice resonance using arrays of half-wavelength gold nanoantennas, achieving a resonance quality factor above 200 [Figure 1(c)] [16]. Using extremely narrow resonance, strong coupling with the vibrational modes of a polymer film coated on the array was observed.

Multiband or broadband resonant MIR nanoantennas are crucial in detecting multiple vibrational modes with large frequency separation. A pioneer study on broadband plasmonic nanoantennas was conducted by Maier’s group using log-periodic trapezoidal nanoantennas for multispectral SEIRA spectroscopy [17]. Recently, Zvagelsky et al. demonstrated broadband SEIRA spectroscopy using Y-shaped nanoantennas by appending a tail to the valley of well-known V-shaped nanoantennas, providing an additional degree of freedom to control the resonance [18]. The Y-shaped nanoantennas were designed to exhibit four resonances in the MIR regions, covering the multiple vibrational modes of the analyte molecule of interest. In addition, Mackin et al. developed dual resonant nanoantennas consisting of trimer nanogap antennas [Figure 1(d)] for dual-frequency SEIRA measurements [19]. The symmetry-broken antennas led to the splitting of the degeneracy of the plasmon modes, enabling the control of the frequency detuning rate by simply changing the length of one of the three nanorods. The dual-band nanoantenna enables the detection of multiple vibrational modes separated by as large as 550 cm^−1^. From a different perspective, Roelli et al. proposed a dual nanogap antenna possessing double resonance in both MIR and visible regions [20]. IR active-molecular vibrational modes were resonantly excited at the MIR wavelength and coupled through their Raman polarizability to a pump field at the visible wavelength, providing a new molecular detection scheme at the single-photon level.

Recently, chiral plasmons have offered a straightforward approach to enhance the relatively weak circular dichroism (CD) effects of chiral molecules, enabling highly sensitive detection and identification of enantiomers. Several chiral and achiral plasmonic nanostructures have been proposed for plasmon-enhanced CD sensing, but they possess resonance mostly in the visible and near-IR regimes [21], [22], [23], [24], [25]. Recently, several MIR-active plasmonic antennas were implemented toward surface-enhanced IR vibrational CD (VCD) spectroscopy [26], [27], [28]. Knipper et al. designed a chiral plasmonic array comprising gold cross slits in which one slit is oriented by 45° clockwise or anticlockwise with respect to the other slit [26]. Cross slits with different slit lengths provide two different MIR resonances, which are independently excited by controlling the incident polarization. The chiral arrays were coated with a silicon nitride (SiN) membrane, which plays an effective role in transporting analyte molecules into the vicinity of the antennas. SiN-coated chiral antennas were used to measure a racemic mixture of *α*-pinene enantiomers, demonstrating enantiomeric sensing with a strong CD absorption of 0.83 [27]. Similarly, Mattioli et al. designed and fabricated chiral slit pairs arranged in a square lattice, revealing the narrow plasmonic CD line shapes [28]. Recently, Mahmud et al. elucidated the plasmon–photon coupling between the plasmon resonance of chiral plasmonic structures and phonon vibrational mode [29]. They revealed electromagnetically induced transparency (EIT)-like resonance under right-handed circularly polarized light with strong plasmon–phonon coupling, and electromagnetically induced absorption (EIA)-like resonance was observed under left-handed circularly polarized light excitation with weak coupling. Iida et al. also designed super chiral plasmonic nanostructures consisting of an Au nanorod pair with a longitudinal displacement [30] as shown in Figure 1(e). The left- and right-handed arrays selectively excited super-chiral fields at 1600 cm^−1^ under linearly polarized light, demonstrating high-sensitive vibrational CD spectroscopy of the mirror-symmetric CD spectra of D- and L-alanine.

The above-mentioned MIR plasmonic antennas were obtained mainly using gold-based nanostructures because gold exhibits high-Q resonance in the MIR region as well as high chemical stability. Aluminum (Al) is also a promising MIR plasmonic material, which has been successively employed in SEIRA spectroscopy [31], [32], [33], [34]. Although Al is naturally oxidized at ambient, the oxide layer is an efficient protecting layer against sulfur contamination and improves chemical functionality. In practice, Al is more cost effective than other plasmonic materials, providing cost effective plasmonic chips. Nagao’s group conducted pioneer studies on MIR-resonant Al nanostructures, validating the great potential of Al as an alternative plasmonic material for SEIRA spectroscopy [31], [32], [33].

### 2.2 Dielectric nanostructures

**Figure 2:**
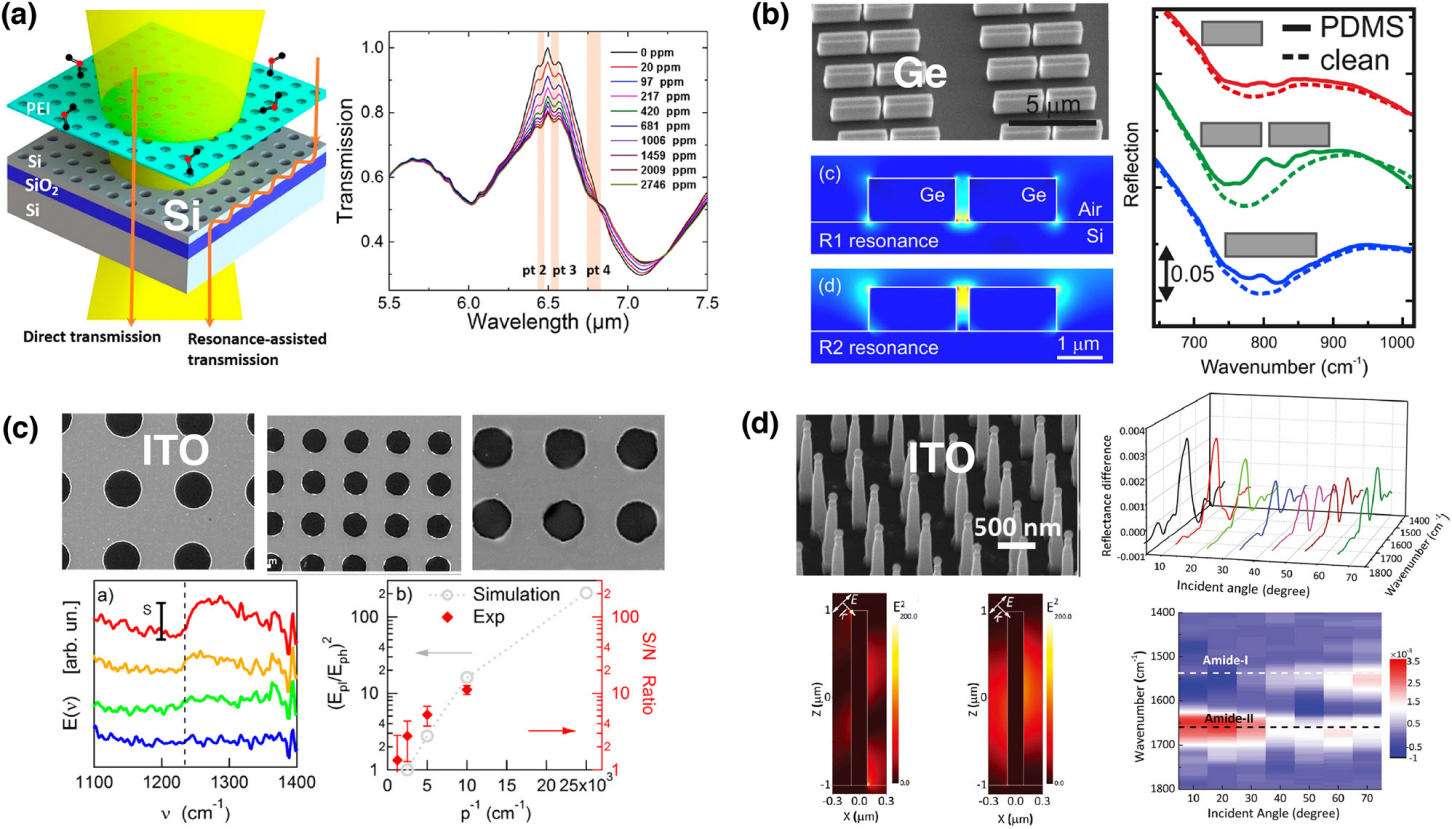
Dielectric nanostructure-based SEIRA structures. (a) Si photonic crystal slab: schematic illustration of the slab and its application to highly-sensitive SEIRA sensing of CO_2_ gas. (b) Highly-doped germanium nanogap antenna: SEM image, calculated electric field distribution at two resonant frequencies, and SEIRA spectra. (c) Microperiodic ITO hole arrays: SEM images of arrays with various lattice periods, their SEIRA spectra and field enhancements. (d) Periodic arrays of vertically aligned ITO nanorods: SEM images and near-field intensity profiles at two resonant modes (1500 cm^−1^ and 2500 cm^−1^). The reflectance difference spectra of the streptavidin molecules on ITO nanorods under different incident angles are also shown. [(a) Reproduced with permission from reference [35]. Copyright © 2018, American Chemical Society; (b) reproduced with permission from reference [44]. Copyright © 2015, American Chemical Society; (c) reproduced with permission from reference [49]. Copyright © 2018, American Chemical Society: (d) reproduced with permission from reference [46]. Copyright © 2017, WILEY-VCH.]

All-dielectric nanostructures with a high refractive index have recently emerged as an alternative to plasmonic nanostructures for highly sensitive SEIRA spectroscopy. Dielectric nanostructures provide high field enhancement and confinement in their proximity owing to their high indices. Another crucial aspect of resonant dielectric nanostructures arises from the low optical loss in the MIR region, resulting in a much higher quality (*Q*) factor than plasmonic structures. Among several dielectric materials, Si and germanium (Ge) are the most widely used dielectric material owing to their high indices and extremely low loss in the MIR region. Chang et al. designed Si-based photonic crystal slabs (PCS) for SEIRA spectroscopy, which exhibit a much higher *Q* factor than Si nanodisks [35]. PCS provides guided resonance-induced enhancement in the effective path length and electric field, achieving a detection limit of 20 ppm in CO_2_ sensing [Figure 2(a)]. In addition, metamaterial-based all-dielectric nanostructures are promising candidates to further improve the *Q* factor [36, 37]. High-*Q* resonance, which is designed to be narrower than target molecular vibrational bands, enables the monochromatic SEIRA sensing of a specific molecular analyte of interest without using an IR spectrometer. Recent studies on all-dielectric metamaterials for SEIRA spectroscopy are highlighted in the latter part of this review article.

Heavily-doped semiconductors are also promising MIR resonators for SEIRA as they exhibit plasmonic behavior [38]. The resonant wavelengths can be tailored to cover the MIR region by doping and/or carrier injection [39], although high doping concentrations are required for MIR resonance. Doped semiconductors also benefit from well-established semiconductor technologies in terms of fabrication and synthesis, resulting in the high-quality and low-cost fabrication of resonant nanostructures. The first application of doped semiconductors in SEIRA spectroscopy was demonstrated in 2013 using Si-doped indium arsenide (InAs) nanoislands [40]. Since then, several triple combinations (dopants, semiconductors, and antennas), such as doped InAsSb ribbons (i.e., gratings) [41] and nanoantenna arrays [42], F- and Sn-codoped In_2_O_3_ cubic nanocrystals [42], and Si-doped InAs ribbons [43], have been proposed for SEIRA-active nanostructures. Baldassarre et al. used heavily-doped Ge nanogap antennas on Si wafers, enhancing molecular absorption at the nanogap by two orders of magnitude [44] as presented in Figure 2(b).

Transparent conducting oxides (TCOs) are oxide plasmonic materials in the visible and near-IR regions [45], but they can also exhibit oxide plasmon resonances in the MIR region if their structures are carefully engineered [46, 47]. Indium tin oxide (ITO) is a promising TCO material as it possesses a considerably high density of free charge carriers. In contrast to gold nanoantennas, ITO nanoantennas do not couple with neighboring antennas in their arrays due to extremely small scattering, increasing the density of nanoantennas as much as possible without plasmonic perturbation [48]. D’apuzzo et al. introduced microperiodic hole arrays into an ITO thin film highly suitable for SEIRA [49] [Figure 2(c)]. By decreasing the lattice period from 8 to 2 µm, the ITO arrays entered the plasmonic regime from the photonics, enabling the detection of SEIRA of polymethyl methacrylate (PMMA) with a high S/N ratio of ∼10. Chen et al. fabricated single-crystalline ITO nanorod arrays [46]. The resonant wavelength and intensity were tuned by varying the incident angle [Figure 2(d)], which allowed selective enhancement of the two amide bands from streptavidin. ITO-based nanoantennas do not require high-cost nanoscale fabrication; hence, they provide a practical way to produce inexpensive sensing devices.

### 2.3 Graphene and van der Waals nanostructures

**Figure 3:**
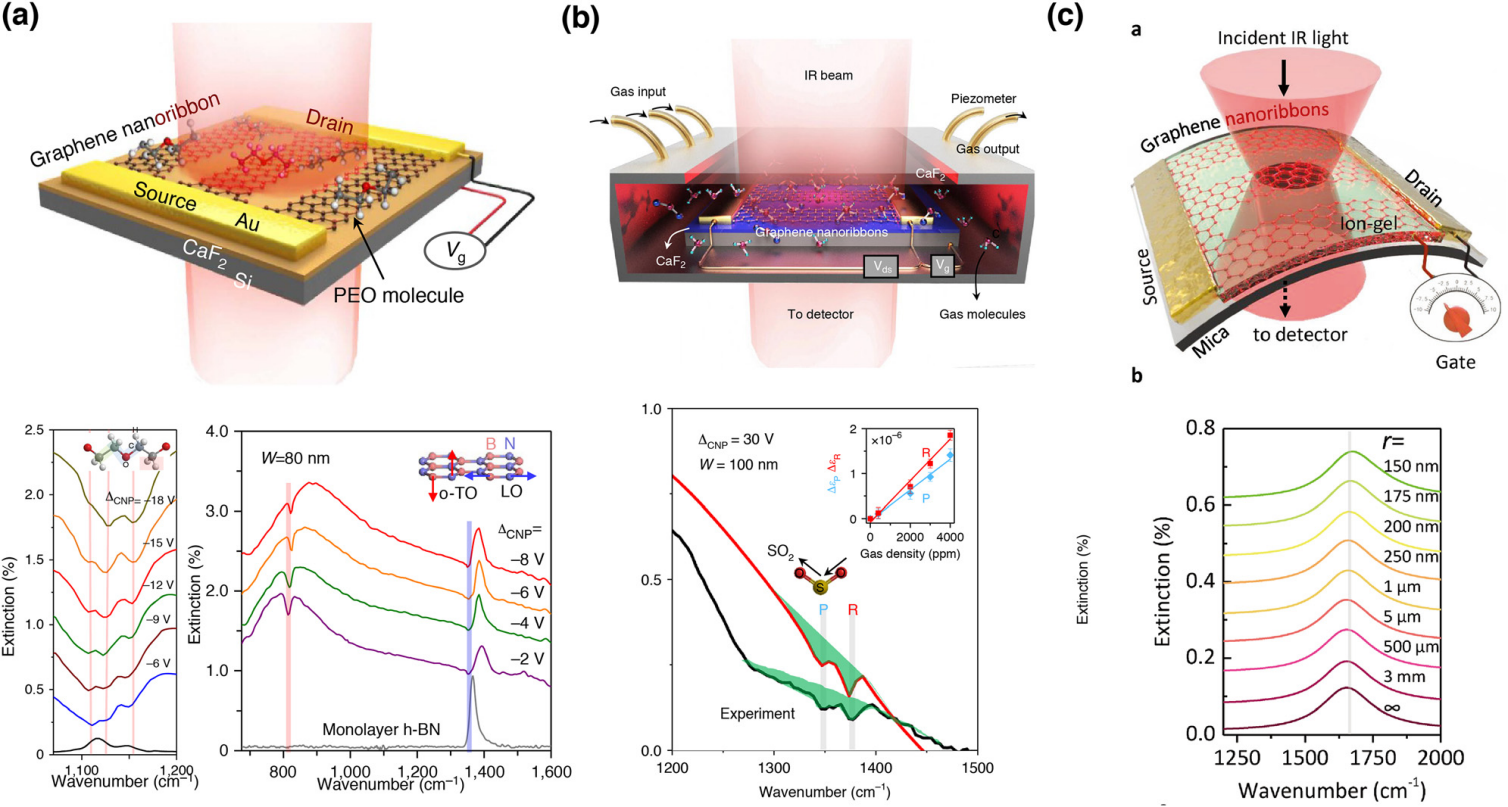
Graphene nanoribbon-based SEIRA structures. (a) A schematic of the sensor and electrically-controlled SEIRA spectra of polyethylene oxide (PEO) thin film and h-BN monolayer. (b) A schematic of the gas sensor and SEIRA spectra of SO_2_ molecules. (c) A schematic of the flexible sensor and calculated extinction spectra of free-standing graphene plasmon on arc nanoribbons bending. [(a) Reproduced with permission from reference [52]. Copyright © 2016, Nature Publishing Group; (b) reproduced with permission from reference [57]. Copyright © 2019, Nature Publishing Group. (c) Reproduced with permission from reference [60]. Copyright © 2018, WILEY-VCH.]

Graphene-based nanostructures are promising SEIRA-active nanoantennas owing to their extreme field confinement originating from the unique electronic band structures of graphene [50]. Graphene plasmon resonances can emerge in the MIR region by introducing periodic subwavelength patterns, such as arrays of nanoribbons [51, 52], nanoholes [53], nanoslits [54], and nanodisks [55, 56], on graphene surfaces, which maximize the near-field enhancement. MIR resonance can be tuned over the MIR fingerprint region by modulating the carrier density (i.e., the Fermi level) of graphene via electrostatic gating and chemical doping. Hu et al. used MIR-resonant graphene nanoribbons to demonstrate *in situ* electrical tuning of graphene plasmons over the entire fingerprint region [Figure 3(a)] [52]. The highly confined graphene plasmons enabled extremely high detection sensitivity to the submonolayer level. Using strong near-field components in the direction perpendicular to graphene, the authors detected out-of-plane and in-plane vibrational modes, which cannot be achieved using typical Fourier transform infrared (FTIR) measurements. They also used graphene nanoribbons for label-free identification of gas molecules adsorbed on the graphene surface, detecting a concentration of 800 zmol per μm^2^ [Figure 3(b)] [57].

Recently, Nong et al. explored graphene plasmons of multilayer graphene nanoribbons [58]. By incorporating a Fabry–Perot-like cavity, they achieved significant improvement in localized graphene plasmonic absorption from 3% to more than 92% and a maximum SEIRA enhancement factor of 162, which is one order of magnitude larger than that obtained using monolayered graphene nanostructures. In addition to the electromagnetic enhancement of graphene plasmons, Hu et al. achieved pH-dependent SEIRA enhancement of aromatic molecules adsorbed on graphene via *π*–*π* stacking interactions, indicating a chemical enhancement similar to that of graphene-enhanced Raman scattering in the visible region [59]. Recently, Hu et al. found that graphene nanoribbons exhibited strain-independent plasmon resonances as shown in Figure 3(c). The resonance frequency, *Q* factors and electrical tunability did not degrade at all even after 1000 bending cycles at a bending radius of 3 mm, opening a new window for flexible active nanophotonic devices [60].

Besides two-dimensional (2D) materials, Autore et al. used hexagonal boron nitride (h-BN), a van der Waals material, for SEIRA spectroscopy. They fabricated h-BN nanoribbons working as hyperbolic phonon–polariton (HPhP) resonators in the strong coupling regime, achieving highly sensitive detection of thin monolayers of the organic semiconductor, 4,4′-bis(N-carbazolyl)-1,1-biphenyl (CBP) [61]. Recently, the same group extended their study to launch HPhPs in thin h-BN slabs using resonant plasmonic antennas [62].

## 3 Metamaterial-based SEIRA

### 3.1 Metamaterial absorber for SEIRA

**Figure 4:**
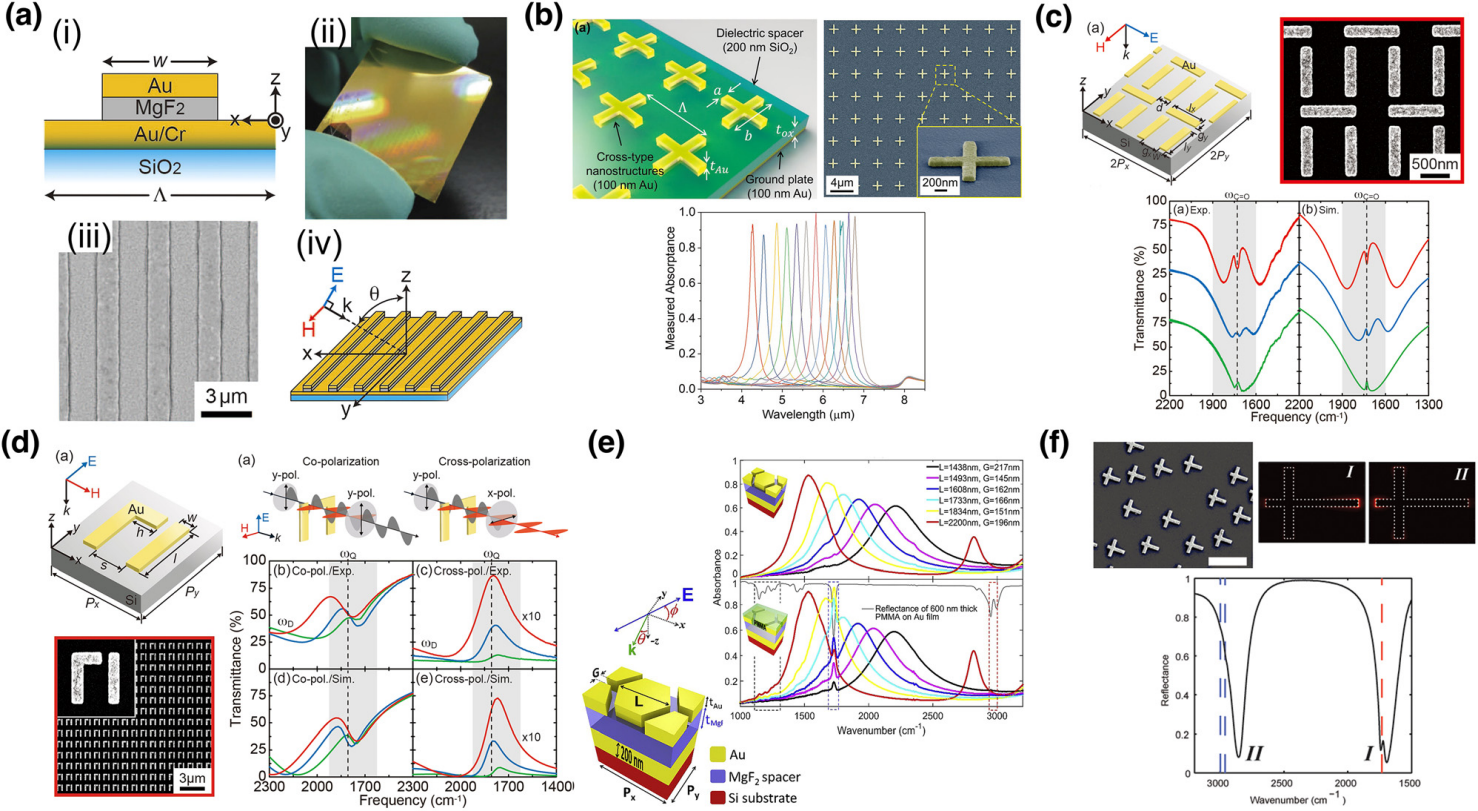
Metamaterial based resonant SEIRA techniques. (a) Resonant SEIRA substrate using MIM metamaterial absorber made of Au and MgF_2_ (b) cross-shaped MIM IR metamaterial absorber for ultranarrow absorption band. Its 3D schematic and scanning electron microscope (SEM) image with absorption spectra. (c) Schematic of super-cell of an EIT metamaterial consisting of two Au bars coupled with Au bar pairs and its SEM image. Experimentally obtained and numerically calculated spectra are also shown. (d) Design and SEM image of fabricated Fano-resonant asymmetric metamaterial for cross-polarized SEIRA, and experimental setups for co- and cross-polarized FTIR measurement with experimentally obtained and calculated spectra. (e) The schematic of honeycomb-shaped metamaterial-plasmonic absorber and experimental result of triple band detection of PMMA thin film. (f) SEM image of planar composite monopole antenna structure, enhanced near-field distribution, and SEIRA spectrum of PMMA are shown. [(a) Reproduced from reference [65] with permission from Ishikawa et al.; (b) reproduced from reference [66]. © 2018 WILEY‐VCH Verlag GmbH & Co. KGaA, Weinheim; (c) reproduced from reference [67]. Rights managed by AIP publishing; (d) reproduce from reference [69] with permission from Ishikawa et al.; (e) reproduced from reference [70]. © 2019 Elsevier B.V. All rights reserved. (f) Reprinted from reference [72] and [73]. Copyright © 2011, American Chemical Society and Copyright © 2012, American Chemical Society]

Metamaterials are artificial optical materials consisting of subwavelength structures. By engineering such structures, unprecedented optical properties and functionalities have been achieved. In vibrational spectroscopic techniques, the most important functionality achieved by metamaterials is perfect absorption. In 2008, a perfect metamaterial absorber structure was proposed by Landy et al. in the GHz frequency region [63]. The proposed structure consists of an electric ring resonator and a cut wire pair separated by a dielectric substrate. Electrical coupling was achieved using an electric ring resonator, resulting in an Ohmic surface loss. On the other hand, magnetic coupling was achieved by a combination of the electric ring resonator and cut wire structures, resulting in a dielectric loss. With the formation of both Ohmic and dielectric losses, nearly perfect absorption was realized using such a simple structure. Recently, this structure is termed metal–insulator–metal (MIM) structure. Compared to the broad absorption band of metal nanostructures, using metamaterial absorbers, almost perfect absorption can be realized. The first IR metamaterial perfect absorber was obtained in 2010 by Padilla et al. using a cross-shaped Au resonator with an Au ground layer separated by an Al_2_O_3_ spacer [64]. A simple MIM structure consisting of Au microribbon on a thick Au film separated by a magnesium fluoride (MgF_2_) spacing layer was introduced to the SEIRA of self-assembled monolayers (SAM) as shown in Figure 4(a) [65]. SAM of 16-mercaptohexadecanoic acid was deposited on the surface of MIM. By FTIR measurements, symmetric and asymmetric C–H stretching vibrational modes were observed as Fano-like antiresonance peaks at the bottom of the absorption band of the metamaterial absorber.

Ultranarrow-band MIM metamaterial absorbers have been demonstrated using a periodic array of cross-shaped Au nanostructure with continuous ground plane separated by a thin SiO_2_ layer [Figure 4(b)] [66]. Capacitive coupling between neighboring unit structures forms narrow absorption bandwidth of 180 nm at a wavelength of 5.83 µm and near-perfect absorption simultaneously.

The surface density of hot spots over a sample is crucial to trap molecules to contribute to the SEIRA signals. Figure 4(c) shows a staggered aligned metamaterial absorber. It enables the full control of plasmonic interference, and a highly dispersive dense quadrupole mode was excited, giving a stronger molecular signal [67].

Due to the intrinsic Ohmic losses in noble metals, such as Ag and Au, it is difficult to realize a sharp resonant absorption (high-*Q* factor); even the separation of amide vibrations (amide I and amide II) requires a *Q* factor of ∼10. To overcome this limitation and provide higher *Q*, a plasmonic Fano-resonant asymmetric metamaterial structure, an array of two parallel Au nanobar structures and a perpendicular antenna coupler attached to one of them, was proposed [68]. To improve the sensitivity of metamaterial-based SEIRA by suppressing the unwanted signal background, a cross-polarized detection scheme has been introduced to Fano-resonant asymmetric metamaterials [Figure 4(d)] [69]. Owing to the elimination of unwanted backgrounds, the S/B ratio is greatly enhanced, and the sensitivity of SEIRA is improved to the zeptomolar level.

### 3.2 Multiband metamaterials for broadband detection

Honeycomb-shaped Au nanostructures produce triple-band metamaterial absorbers [Figure 4(e)] [70]. The honeycomb geometry creates three absorptive bands, which cover the 1150–1250, 1732, and 2952–2992 cm^−1^ regions, corresponding to the C–O–C antisymmetric stretching mode, C=O stretching mode, and stretch combination mode of C–H, respectively. Using this structure, multiband SEIRA detection was conducted using a 10-nm-thick PMMA film. Fan and rectangle pad-pod nanoantenna arrays also provide multiple absorption bands [71]. This structure can also be used for SEIRA measurement of C=O (1732 cm^−1^) and C–H (2955 and 2989 cm^−1^) signals of PMMA.

Asymmetric cross-shaped nanoantenna structures [Figure 4(f)] work as a composite double-monopole antenna and provide dual absorption bands determined by the arm length. A wire reflector acts as a ground plane to isolate the antenna and offers electrical isolation between conductively coupled antennas. It also allows us to circumvent the necessity of three-dimensional MIM structures. Using four arm-length monopole antennas, the SEIRA signals of amide I and II were detected [72]. Dual-band perfect absorber structures based on asymmetric cross structures with an additional Au thin film and an MgF_2_ spacing layer showed two absorption bands up to 94%, and they are used for multiband SEIRA. By changing the arm length, two absorption bands are tuned to the absorption frequency of the C–H and C=O absorption bands of PMMA, which are separated by 1200 cm^−1^, demonstrating the simultaneous detection of these two vibrational signals [73].

## 4 Advanced functionalities

### 4.1 Absorption band tuning

**Figure 5:**
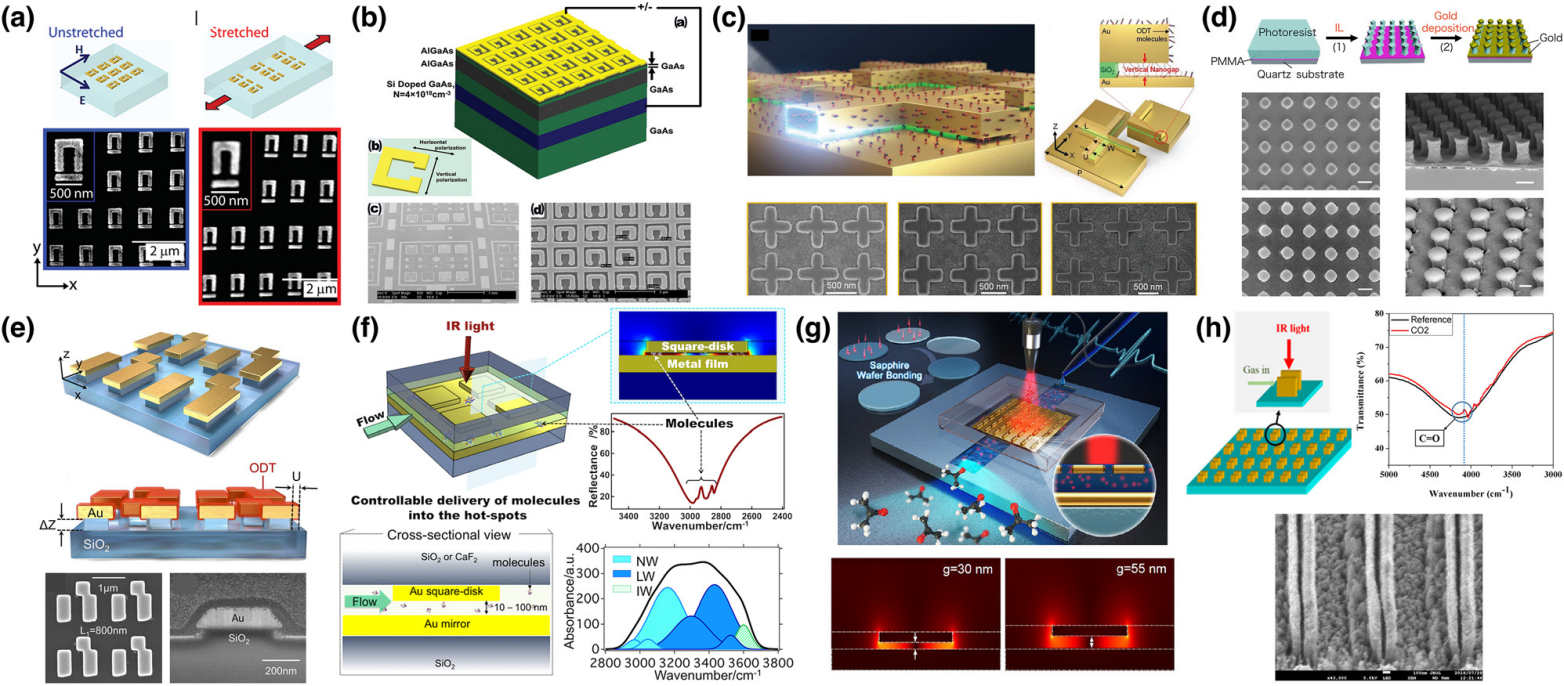
Metamaterial-based SEIRA structures with advanced functionalities. (a) The schematics and SEM images of coupled Au SRR-bar structure fabricated on PDMS substrate before and after substrate stretching. (b) Electrically tunable mid-infrared metamaterial structure made of AlGaAs/GaA with its SEM images. (c) The schematics of the infrared metamaterial absorber with vertical nanogap and its unit structure. Top view of SEM images of different vertical nanogap structures is also shown. (d) Fabrication procedure and SEM images of fabricated gold mushroom array structure. (e) The schematics of the Fano metamaterial structure on the SiO_2_ nanopedestal with top and cross-sectional SEM images. (f) The schematic of metamaterial-nanofluidics hybrid structure for ultrasensitive platform for SEIRA. Retrieved vibrational modes of water measured by the device are also shown. (g) Self-driven Al_2_O_3_-based 3D plasmonic and nanofluidic hybrid device and its simulated electrical field distributions. (h) Vertical MIM metamaterial structure for gas sensing application and its SEM image. SEIRA spectrum of carbon dioxide is also shown. [(a) Reproduced from reference [74]. Copyright © 2010, American Chemical Society; (b) reproduced from reference [76]. Rights managed by AIP Publishing. (c) Reproduced from reference [79]. © 2021 The authors. Small methods published by Wiley-VCH GmbH; (d) reproduced from reference [80]. Copyright © 2013, Nature Publishing Group, a division of Macmillan Publishers Limited. All Rights Reserved; (e) reproduced from reference [81] Copyright © 2019, Jung et al.; (f) reproduced from reference [82] and [24]. Copyright © 2017, American Chemical Society and Copyright © 2018, American Chemical Society; (g) reproduced from reference [85]. Copyright © 2020, American Chemical Society; (h) reproduced from reference [87]. Copyright © 2019, American Chemical Society.]

In conventional nanostructures, their optical properties, including resonant absorption wavelengths, are fixed once the structures are fabricated. In practical applications of SEIRA, there is a need for *in situ* tunability of the resonance over the vibrational modes of the sample molecules. Precise tunability of resonant frequency was introduced using an array of coupled SRR–bar structures fabricated on a soft polydimethylsiloxane (PDMS) film as shown in Figure 5(a) [74]. After transferring Au SRR–bar structures to the PDMS film and stretching the PDMS film, the structure was deformed to a particular distance between SRR and bar was changed, resulting in the resonant frequency change. In the experiment, the intensity of the electric field near the structure on PDMS is an order of magnitude weaker than that of the structure on the solid Si substrate because of the low refractive-index contrast of PDMS compared to that of Si. However, the PDMS system could demonstrate the tunability of the resonant frequency by post-stretching treatment, and the resonant wavelength was permanently red-shifted from 5.78 to 6.27 µm. This strained compliant metamaterial structure was also employed for refractive-index sensing, and it showed an FOM value of up to 10.1 [75].

Electrical modulation of the optical properties of MID metamaterials has been demonstrated using an SRR array on an AlGaAs/GaAs Schottky diode structure [Figure 5(b)] [76]. By applying a bias voltage to the diode, a 10% wavelength shift and a 10% transmission change at a wavelength of 10 µm were obtained, corresponding to the fundamental resonant wavelength of split ring resonators.

Incident-angle tuning techniques for resonant frequency on metal-rod array structures in the mid-IR region have been proposed. By changing the incident angle in FTIR measurements, an enhanced frequency of the metal-rod array structure was spectrally tuned by approximately 400 wavenumbers, which covers the spectral range of symmetric and asymmetric CH_2_ vibrational modes of 16-mercaptohexadecanoic acid (16-MHDA) [77].

### 4.2 Further improvement of sensitivity by increasing interactions between metamaterial and molecules

To further enhance SEIRA signals, increasing near-field coupling with the IR absorption mode of the sample molecules is crucial. Lifting metamaterial structures above the substrates is a practical and effective way to enhance SEIRA and surface plasmon sensors because it exposes an enhanced electric field to the space, becoming easily accessible by analyte molecules. A rectangular Au patch array structure has been fabricated on a Si substrate by electron beam (EB) lithography. Employing selective and isotopic ion etching through inductive-coupled plasma reactive ion etching (RIE) with zero forward power, the Si substrate underneath an Au pattern was also etched, and the Au pattern was elevated from the surface of the Si substrate [78]. As shown in Figure 5(c), a vertical nanogap was formed between the top nanoantenna and bottom ground plane by undercutting an extremely thin (10 nm) dielectric spacer layer using an isotropic dry etching technique, inducing an extremely strong electromagnetic field between the top and bottom metal layers and extending the effective interaction area of the light wave and molecules. Using this device, 36% reflection difference in the SEIRA signal of a 1-octadecanethiol monolayer at a wavelength of 3.5 µm was demonstrated [79].

A gold mushroom array (GMRA) structure, which consists of an Au cap on a dielectric pillar, forming a mushroom-like structure [Figure 5(d)], was proposed by Shen [80]. Double two-beam interference lithography was used to form a periodic array of photoresist pillars, and an Au thin film was deposited by thermal evaporation to form GMRA. Owing to the narrow full-width at half maximum (FWHM) plasmon resonance and large accessible area for plasmon-enhanced electric fields around the Au caps, large FOM (up to 108) and low detection limits [down to 200 pM for cytochrome c (Cyto c) and 15 ng ml^−1^ for alphafetoprotein (AFP)] have been experimentally demonstrated. The same idea was introduced to Fano-resonant metamaterial structures. Fano metal structures consisting of two parallel Au nanoantennas with a perpendicular antenna coupler attached to one of them have been fabricated by nanoimprint patterning and isotropic dry etching and used to undercut SiO_2_ substrates forming dielectric pedestal [81]. Three FMM structures with different antenna lengths were fabricated and used for SEIRA measurement of a 1-octadecanethiol monolayer deposited on the surface of the device. More than 7% of the reflection difference SEIRA signal, which is 1.7 times higher than that of the control (without pedestal) structure, was obtained.

Since SEIRA signals can be obtained when the analyte molecules are spatially introduced into the nanoscale hotspot regions of MIM structures, efficient usage of hotspots and precise control of analyte molecules to their region are crucial. Nevertheless, in conventional MIM-based MPA analyte molecules are deposited on the surface of the top plasmonic structure, and only small parts of the enhanced field are utilized. To solve this problem, a nanofluidic channel with a depth of several tens of nanometers was introduced to a plasmonic MIM structure as shown in Figure 5(e), and it delivers analyte molecules to the hotspot region, which is the most enhanced field obtained from the quadrupole mode of MIM structures [82]. Owing to the controllable delivery of analytes into the hotspot region, the asymmetric and symmetric stretching modes of –CH_2_ bond of octadecane (C_18_H_38_) in CCl_4_ solution were clearly detected. This method was employed to characterize the structure of molecules confined in a space of several tens of nanometers. Water was used as a test sample and introduced to the nanofluidic channel. The O–H stretching band of the confined water was selectively detected, and it could elucidate and identify the differences in the molecular structures and refractive index of water confined in spaces of several tens of nanometer [Figure 5(f)] [83,84]. The optical properties of the plasmonic and nanofluidic hybrid device were systematically studied, and vertically enhanced hotspots, which areformed at the fluidic channel, enable higher sensitivity compared with the dipole plasmons at the top metal layer [85]. A similar structure consisting of Au cut wire array and planar Au thin film on a Si substrate with nanometer gap spacing has been reported, and introducing liquid samples using capillary force, SEIRA spectra of liquid samples, such as acetone, ethanol, isopropyl alcohol, and methanol, can be investigated [86].

To introduce analyte molecules into the hotspot region formed inside an insulation layer sandwiched by two metal plate in the case of MIM structures and extend the accessible space of analyte molecules to the enhanced near-field near metamaterial unit structures, a vertical MIM (v-MIM) structure shown in Figure 5(g) was proposed [87]. Owing to the small footprint of v-MIM, the density of MIM structures can be easily increased, further enhancing SEIRA signals. In the experiment, the C=O vibration mode of CO_2_ and symmetric/asymmetric stretch absorption bands of butene were obtained.

## 5 New materials and methods

### 5.1 Dielectric metamaterial SEIRA

Noble metals, such Ag and Au, are more commonly used in this field, and their plasmonic properties have been studied [88]. Intrinsic optical losses of noble metals cause inevitable and unwanted degradation of the *Q* factor of metal-based metamaterials, limiting the field enhancement efficiency and sensitivity of SEIRA. Recently, as an alternative to noble metals, nonplasmonic materials were investigated for SEIRA applications. All materials have their advantages and drawbacks, which have already been summarized in ref. [89].

**Figure 6:**
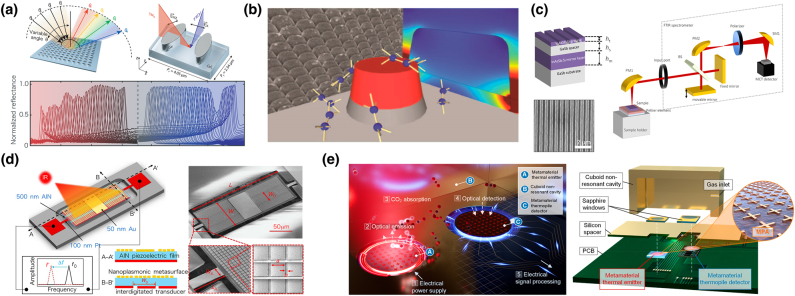
Metamaterial structure with new material and for new methods. (a) The schematics of all-dielectric metasurfaces for angle-multiplexed broadband molecular detection and its experimental result of reflection spectra of PMMA thin film. (b) Si-doped InAs semiconductor nanoantenna array for SEIRA. (c) The schematics of Si:InAsSb metamaterial absorber and its SEM image. The optical setup for surface-enhanced thermal emission spectroscopy was also shown. (d) The schematic and SEM image of plasmonic piezoelectric nanomechanical resonant infrared detector. (e) The schematics of metamaterial gas sensor device. Metamaterial thermal emitter and metamaterial thermopile detector were integrated inside nonresonant cavity. [(a) Reproduced from reference [91]. © The Authors, some rights reserved; exclusive licensee AAAS. Distributed under a CC BY-NC 4.0 license http://creativecommons.org/licenses/by-nc/4.0/; (b) Reproduced from reference [40]. Copyright © 2013, American Chemical Society; (c) reproduced from reference [34]. Copyright © 2019, American Chemical Society; (d) reproduced from reference [96]. Copyright © 2016, Hui et al.; (e) reproduced from reference [38]. Copyright © 2020, American Chemical Society.]

Hydrogenated amorphous Si (a-Si:H) was used as a nonmetal metamaterial absorber. A zigzag array of elliptic Si patch array was fabricated in an area of 100 µm × 100 µm on a MgF_2_ substrate as a metapixel. A metapixel has a narrow and strong reflectance band according to its structure [90]. Changing the scaling factor, 100 metapixels with different resonant reflection peaks formed a 10 × 10 array, and it was observed using an MIR microscope. Introducing analyte molecules to the surface of the device, its absorption spectrum was obtained as an envelope of metapixel reflectance amplitudes. A similar structure comprising Ge [Figure 6(a)] was proposed by the same group [91]. Resonant reflection wavelength can be tuned by scanning the incident angles, and using both TE and TM resonant modes, the device could cover a broad spectral range (1100–1800 cm^−1^). Strong field enhancement more than 6000 and its near-field distribution originating from high-*Q* resonance are also shown.

Low-loss surface phonon–polariton modes excited in a polar dielectric material were proposed to tune the resonant spectrum of metasurfaces, and they are used for SEIRA [92]. Also, 4H–SiC has been used, and its nanopillar array structure was fabricated on SiC substrates. In the experiment, broadband spectral tuning over a 60^–^800 cm^−1^ range was demonstrated.

Since doped semiconductors do not have strong interband absorption near their plasma frequencies, they have strong resonance in the IR region. A Si-doped InAs nanoantenna array was fabricated on a GaAs substrate using nanosphere lithography and ion milling techniques [Figure 6(b)]. The surface of the device was coated by a 50-nm-thick PMMA layer as a test sample, and its absorption lines were observed [40].

Heavily Si-doped indium arsenide antimonide (InAsSb:Si) shows metal-like properties in the mid-IR spectral regime. Using the grating structure of InAsSb:Si, an InAsSb:Si back reflector and a GaSb spacer layer were fabricated on a GaSb substrate [Figure 6(c)]. This structure works as a perfect absorber. Barho et al. used this semiconductor metamaterial absorber as a surface-enhanced thermal emitter [93, 94]. The surface of the device was functionalized with 11-pentafluorophenoxyundecayltrimethoxysilane (PETMS), and its IR spectrum was measured using FTIR. This technique is termed surface-enhanced thermal emission spectroscopy (SETES).

A dielectric grating stripe was proposed for angle-tunable narrowband absorbers. CaF_2_ was used for grating fabricated on a thick aluminum film [95]. Owing to the low absorptivity of CaF_2_, the structure shows a narrow absorption band with an FWHM of 29 nm, corresponding to a *Q* factor of 212. In addition, by varying the incident angle from 0° to 10°, the absorption band at 6.134 µm was split, and one resonant peak was blue-shifted to 5.25 µm, and the other one was red-shifted to 6.80 µm.

### 5.2 Metamaterial IR emitter and detector

A plasmonic piezoelectric metasurface was produced as a spectrally selective IR detector. An Au metamaterial absorber consisting of an Au rectangular patch array was fabricated on AiN piezoelectric self-suspended thin film as shown in Figure 6(d). When IR light illuminates the device, it is selectively absorbed by the metasurface, and the device temperature rapidly increases. An increase in the device temperature changes the mechanical resonant frequency of the piezoelectric film. Thus, the intensity of IR light can be detected by monitoring the resonance frequency [96]. A cross-shaped metamaterial absorber was used as wavelength-selective IR emitters and detectors [97]. All-metamaterial devices and nonresonant cavity were integrated into 5.7 mm × 5.7 mm × 4.5 mm, forming a compact MIR gas sensor [Figure 6(e)].

## 6 Nanoscale chemical imaging via nanostructure-enhanced IR spectroscopy

Herein, we review the nanoscale chemical characterization of materials by SEIRA-based techniques. In conventional IR spectroscopy, the spatial resolution is limited to several micrometers, and the wavelength-dependent spatial resolution makes it difficult to leverage itself for the chemical characterization of materials. Conventional SEIRAs using metallic or dielectric nanostructures boosts the signal amplitude of IR absorption of molecules; however, it cannot probe IR vibrational information with the nanoscale spatial resolution since, in such enhanced IR spectroscopy, optical signals derived from multiple nanostructures within a diffraction-limited laser spot are detected.

**Figure 7:**
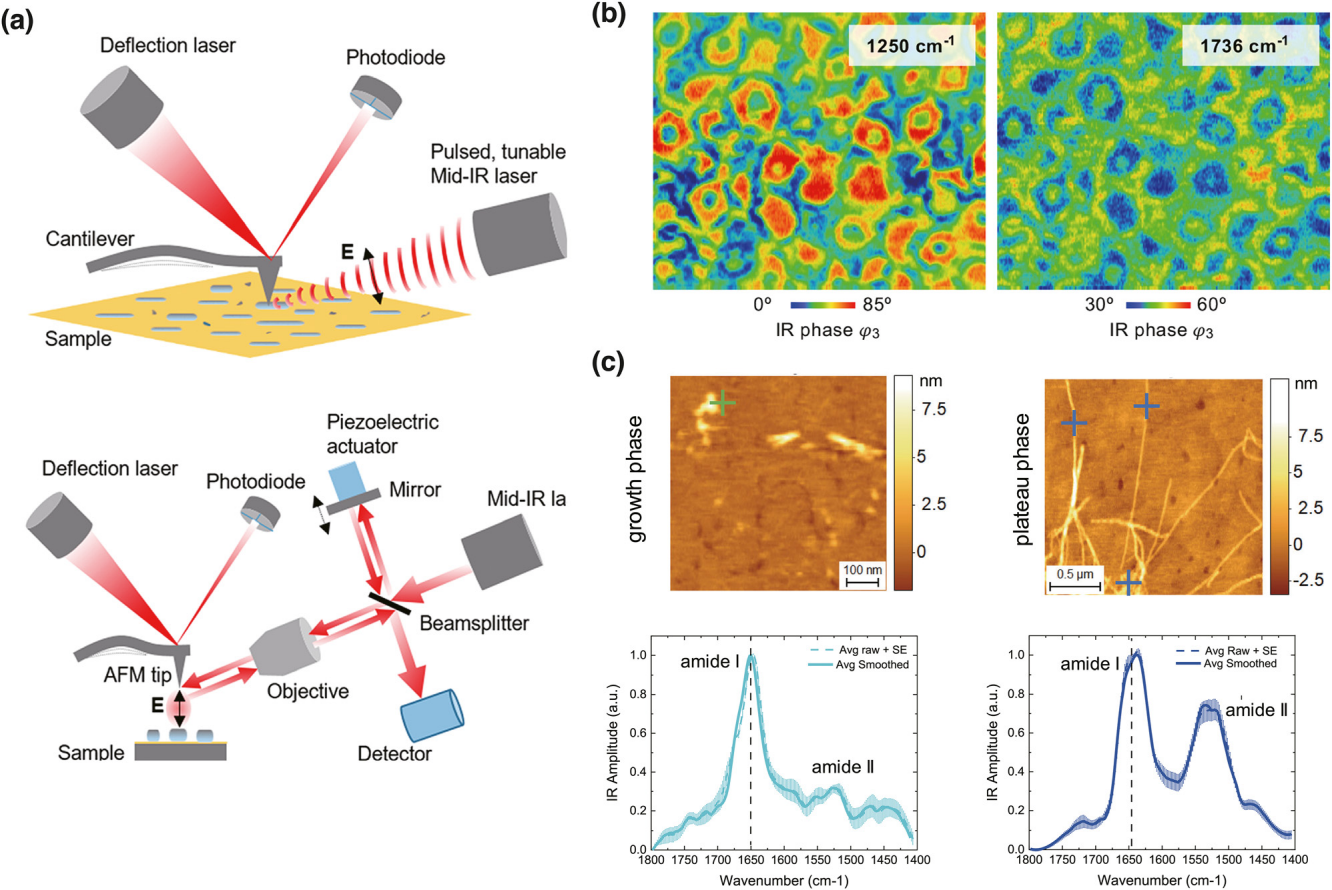
Principles and applications of infrared nanospectroscopy for polymer and biological samples of (a) Schematic of and atomic-force-microscopy-based infrared spectroscopy (AFM-IR) (top) and IR scattering-type scanning near-field optical microscopy (s-SNOM) with an asymmetric Michelson interferometer (bottom). (b) Topography of poly(perfluorooctyl acrylate-co-methyl methacrylate-co-butyl acrylate) copolymer and the corresponding nanoscale IR absorption, constructed by IR peaks at 1250 and 1736 cm^−1^, respectively. (c) Topography and AFM-IR spectra of single oligomeric (left hand side) and fibrillar species (right hand side) of Aβ42. [(a) Reproduced with permission from reference [101]. Copyright © 2020, American Chemical Society; (b) reproduced with permission from reference [127]. Copyright © 2021, American Chemical Society; (c) reproduced with permission from reference [134]. Copyright © 2021, Francesco Simone Ruggeri et al.]

IR scattering-type scanning near-field optical microscopy (s-SNOM) and atomic force microscopy-based IR spectroscopy (AFM-IR) are primary nanoscale IR spectroscopic techniques developed independently. Both techniques combine IR spectroscopy with the modality of scanning probe microscopy (SPM) to achieve nanoscale spatial resolution and high sensitivity. IR s-SNOM was first developed independently by Hammiche et al. [98] and Anderson [99] in 2000, and AFM-IR was first demonstrated by Dazzi et al. in 2005 [100]. Despite sharing the same platform of SPM, signal transduction of IR absorption in both techniques follows fundamentally different principles, as shown in Figure 7(a) [101]. In s-SNOM, a sharp metallic nanotip is brought in the vicinity of a sample and illuminated by the incident IR light. The amplitude and phase of the scattered light on the sample excited by strongly confined and enhanced near-field light at the apex of the metallic tip are measured. These optical signals contain information about the refractive index and absorption coefficient of the sample based on wavelength-dependent IR absorption. On the other hand, AFM-IR mechanically probes the IR absorption of a sample and transduces IR absorption signals using sharp metallic or dielectric tips in the near-field, unlike s-SNOM that optically probes the IR absorption of samples. In AFM-IR, a pulsed wavelength-tunable IR laser source is used to excite the IR absorption of samples. The sample absorbs IR pulsed beam, resulting in heating up and thermal expansion. When the tip is in the vicinity of the sample, the thermal expansion of the sample can be detected by mechanically induced oscillation of the tip. The amplitude of the induced oscillation of the tip is proportional to the local IR absorption of the sample. Hence, detecting the amplitude of the tip while sweeping the pulsed IR laser allows the IR absorption spectrum of the sample to be obtained in AFM-IR. By fixing the laser frequency and raster scanning the sample, an IR absorption image of the sample can be obtained at the nanoscale. In both s-SNOM and AFM-IR, metallic nanostructures fabricated on a nanotip play pivotal roles in enhancing the detection sensitivity of IR absorption signal. When the sample is exposed to plasmonically enhanced optical fields at the apex of the metallic tip, IR absorption of the sample is locally enhanced. The plasmonic enhancement by metallic nanostructures produces high sensitivity of IR nano-spectroscopy, which detects samples only a few nanometers thick or even monolayers [102, 103]. The spatial resolution of IR nanoimaging achieves a few tens of nanometers, depending on the size of the apex of the nanotip [104, 105], and in some cases, it reaches 5 nm in the gap-mode AFM-IR configuration [106]. In this review, the working principles of s-SNOM and AFM-IR are not described in detail, but they can be found in the literature [107, 108]. Numerous applications of SEIRA-based spectroscopy and nanoimaging have been demonstrated in several fields, including polymer science [109], [110], [111], photovoltaics [112, 113], pharmaceuticals [114, 115], biology [116], [117], [118], [119], [120], and plasmonic and nanophotonics [121], [122], [123], [124]. Herein, we highlight recent applications of IR s-SNOM and AFM-IR for characterization of the chemical and optical properties of materials.

### 6.1 Chemical characterization of polymeric samples

The IR nanoimaging techniques have explored the frontiers in polymer sciences since its invention [125]. Polymeric samples, such as polymer blends and block copolymers, have heterogeneous chemical and morphological structures at the nanoscale, which determines the polymer’s performance and functions. Since polymers have many IR active vibrations, nanoscale IR vibrational imaging is a promising way to characterize the chemical properties of polymeric samples to elucidate the mechanism behind their performance and functions.

IR nanoimaging was employed to visualize and identify heterogeneous chemical structures in blend polymers and block-copolymer. Nguyen Tri et al. investigated the chemical compositions of the surface of blend polymer films consisting of two immiscible polymers [polycaprolactone and poly(ethylene glycol)] [126]. They studied the crystallization and phase segregation of polymer blend films through bond-selective chemical nanoscale imaging. Recently, Goikoetxea et al. used IR s-SNOM to show the spatial distribution of multiple chemical compositions in poly(perfluorooctyl acrylate-co-methyl methacrylate-co-butyl acrylate) copolymers [127]. Bond-selective chemical nanoimaging of a copolymer sample composed of methyl methacrylate and butyl acrylate showed the chemical compositions of the inner and outer parts of nanodomains [Figure 7(b)].

Tang et al. visualized the chemical compositions of the nanodomains in commercial high-impact polypropylene (HIPP). They reported that the primary component of the rigid cores of rubber particles in HIPP is polypropylene, whereas polyethylene had been believed as the main component [128]. Morsch et al. also employed AFM-IR imaging in the nanoscale chemical and morphological analysis of epoxy resin and revealed the its heterogeneous chemical compositions [129]. In addition, chemical properties of hybrid materials consisting of polymers and other materials, such as metal oxide [130] and lipids [131, 132] were investigated. Such hybrid materials show remarkable structural and chemical properties, making them promising for efficient drug delivery systems and biological sensing applications.

### 6.2 Applications of IR nanoimaging in life sciences

IR nanoimaging has wide applications in the realm of life sciences owing to the high chemical sensitivity and nanoscale spatial resolution, both of which provide new insight beyond what has been revealed by conventional IR spectroscopy. Since biologically important and relevant functional groups or fundamental building blocks of biological samples, such as DNA and RNA, lipids, and proteins, exhibit characteristic IR vibrational modes in the fingerprint region, IR nanoimaging has been extensively employed to investigate biological functions and phenomena at the nanoscale. For instance, nanoscale chemical imaging via nanostructure-enhanced IR spectroscopy techniques has been applied to cells, lipid droplets, polypeptides, photosynthetic complexes, and lipid membranes. As IR vibrational modes of amide I and II reflect on the secondary structures of peptides and proteins, structural and conformational analysis can be extensively conducted. Ruggeri et al. investigated the aggregation pathway of the Josephin domain of ataxin-3 implicated in spinocerebellar ataxia-3 [133]. Further, *α*–*β* conformational transitions in proteins were monitored at the nanoscale. They also exploited the advantage of the single molecular sensitivity of AFM-IR in investigating the chemical structures of Aβ42 oligomers and studied the aggregation pathway of the oligomers in the presence and absence of an aggregation inhibitor [134]. Figure 7(c) shows topographic images and nanoscale IR spectra of single oligomeric and fibrillar species, respectively. The results suggest that the presence of an aggregation inhibitor induces conformational changes in peptides before and after aggregation. A similar study on the identification of polypeptide conformation at the nanoscale was demonstrated by the Centrone’s group [135]. By discriminating the secondary structures of peptides, it was observed that diphenylalanine and tert-butoxycarbonyl-modified derivative fibrils structurally differ at the individual fibril level.

Biological plasma membranes play a key role in cell life, such as controlling the transportation of ions and molecules involved in various intra- and extracellular processes. Lipid membranes possess inhomogeneous structures where various biomolecules coexist, such as membrane proteins. Therefore, direct visualization of these structures with chemical identification is important. Pioneer studies have been demonstrated by Berweger et al. in 2003, where membrane proteins in lipid bilayers were chemically visualized [136]. Membrane proteins embedded in lipid bilayers were imaged with few-protein sensitivity, which clarified the heterogeneous concentration of proteins in the lipid bilayers. Giliberti et al. confirmed light-induced conformational changes in membrane proteins by AFM-IR [137]. Conformational changes in bacteriorhodopsin associated with its photocycle upon blue or green light were probed by AFM-IR difference spectra. Another type of membrane protein called channelrhodopsin (ChR) was also studied by Temperini et al., and they observed reversible light-triggered conformational changes ChR, even 48 h after the sample was exposed to air. Tremendous efforts have been made on the chemical characterization of polymeric and biological samples using IR nanoimaging, which was reviewed in other review articles [138], [139], [140].

### 6.3 Characterization of the optical field properties of nanostructures

The IR nanoimaging techniques have been employed to explore unique optical properties of plasmonic and photonic nanostructures in the MIR region, including metallic nanorods [141, 142], dielectric gratings [143], and h-BN nanostructures [121, 144, 145]. The strong localization of optical fields in the visible and MIR regions and enhancement of the field intensity lye at the heart of nanostructure-enhanced IR spectroscopy, as discussed in the previous sections. A deep understanding of the plasmonic properties of metallic and dielectric nanostructures, such as the spatial distribution of confined optical fields and the resonance wavelength of nanostructures, in the nanometer scale would contribute to the advancement and optimization of nanostructure-enhanced spectroscopy and its applications. For instance, a detailed understanding of the distribution of optical fields near the metallic or dielectric nanostructures is important to evaluate the sensing capability since the number of molecules interacting with the near-field can be estimated. In most nanostructure-enhanced spectroscopic applications, nanostructures are designed to tune their resonance wavelength at the excitation laser wavelength to maximize the optical field intensity and produce extremely high sensitivity. Hence, nanoscale visualization of the wavelength dependence of localized surface plasmon resonance (LSPR) is imperative to design nanostructures and metamaterials for enhanced spectroscopy in the MIR region.

**Figure 8:**
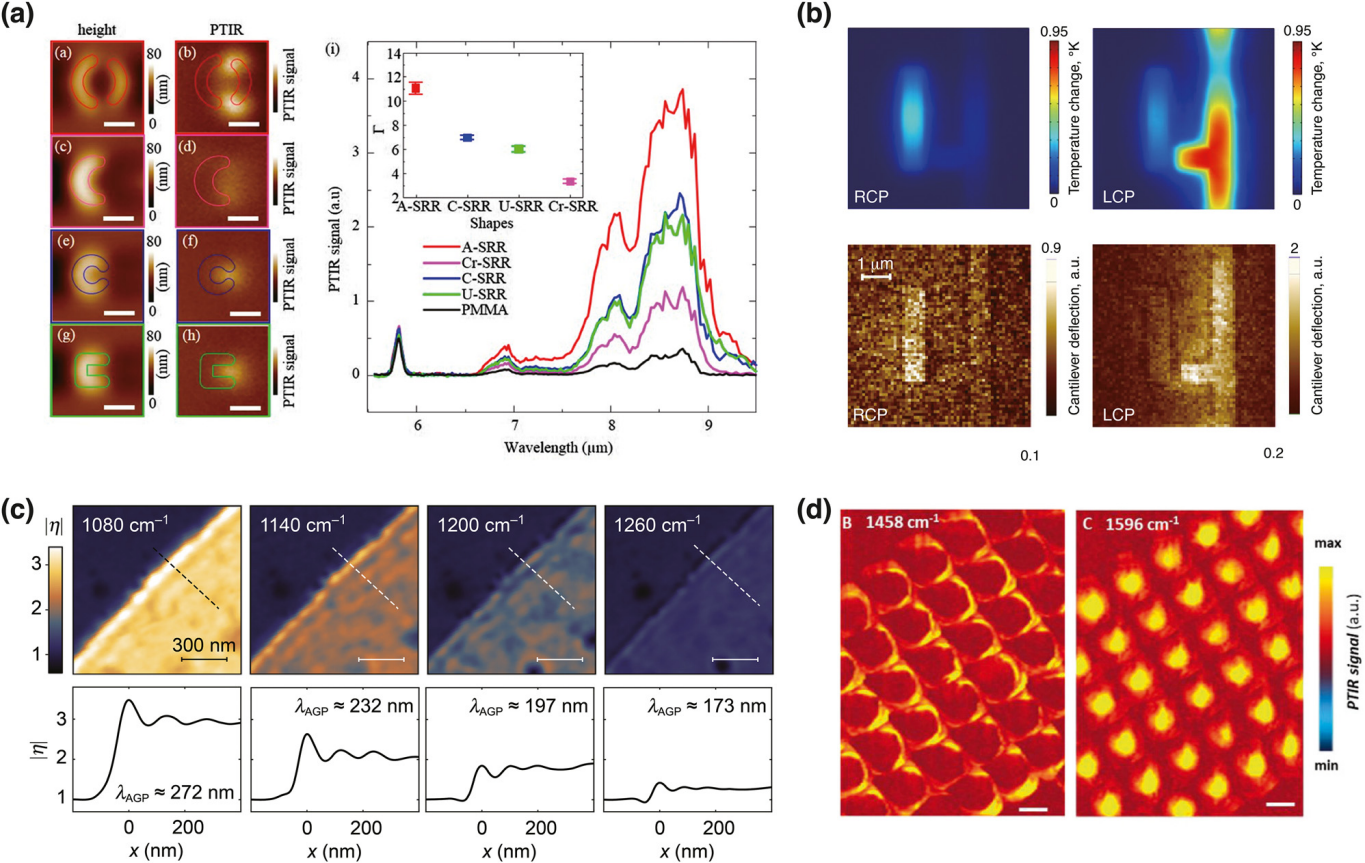
Infrared nanospectroscopy for nanostructures (a) Pairs of simultaneously obtained topography (left) and AFM-IR (right) images of gold resonators with different shapes. The AFM-IR images were constructed by the absorption peak at 1142 cm^−1^, corresponding to the C–O stretching of polymethyl methacrylate (PMMA). The AFM-IR spectra from the hotspot locations in the AFM-IR images are compared with the bulk spectrum of the PMMA film (black). The inset shows the enhancement factors (Γ) of the near-field intensity at 1142 cm^−1^. (b) Simulated distributions of temperature increase in a polyethylene film illuminated by an IR pulsed laser at 9.1 µm, which is the same as the experimental conditions. AFM-IR images of polyethylene-coated metallic chiral nanostructures excited with right circularly polarized (left) and left circularly polarized (right) laser pulses. (c) IR images of the same graphene edge recorded at different frequencies, showing the distributions of polaritonic modes and the corresponding intensity profiles across the edge of graphene along the dashed line in the IR images. (d) AFM-IR map of hexagonal boron nitride structures at 1458 and 1596 cm^−1^. The scale bars are 260 nm for the images. [(a) Reproduced with permission from reference [5]. Copyright © 2016, American Chemical Society. (b) Reproduced with permission from reference [148]. Copyright © 2016, A. B. Khanikaev et al.; (c) reproduced with permission from reference [150]. Copyright © 2021, Sergey G. Menabde et al.; (d) reproduced with permission from reference [154]. Copyright © 2020, Georg Ramer et al., published by De Gruyter.]

The Centrone’s group studies the plasmonic properties of metallic nanostructures using AFM-IR [5, 146, 147]. They proposed a promising experimental technique, where Au nanostructures resonated in the MIR region are coated with polymer films to probe the enhancement of IR absorption since the rapid thermal expansion of metallic nanostructures is difficult due to the small expansion coefficient and large thermal conductivity of metals. Strong enhancement of IR absorption (up to a factor of 28) associated with both bright and dark modes was observed at the nanogap of metallic nanostructures. Further, the authors observed the distribution of the optical field induced by plasmon resonance of Au nanostructures, and the field was confined at the termination of the nanostructures as shown in Figure 8(a). Additional unique optical properties of metamaterials, such as optical activity, can also be investigated. Khanikaev et al. experimentally demonstrated CD in planar-chiral plasmonic metasurfaces, see Figure 8(b) [148]. Nanoscale imaging of optical fields in metasurfaces and enhancement of IR absorption revealed circularly dichroic Ohmic loss in the chiral nanostructures, paving the way for optimal design for SEIRA of chiral biomolecules.

The optical properties of recently emerged nonmetallic nanostructures, such as graphene and h-BN, have also been studied by IR nanoimaging. The plasmonic properties of graphene have been characterized in several studies since the pioneering work reported by Fei et al. in 2012 [149]. Surface plasmons in graphene [150, 151] and nanostructured graphenes, such as twisted graphene [124, 152] and graphene nanoribbons [153] at the IR region, have been characterized, which clarifies the plasmonic behavior in the IR region. Menabde et al. examined acoustic plasmons in graphene using s-SNOM, and they found ultralow loss plasmons. Figure 8(c) shows nanoscale IR images of graphene layers at multiple frequencies and the corresponding line profiles across graphene edges along the dotted line. The present results, for the first time, demonstrated a low propagation loss and the dispersion of acoustic graphene plasmons.

In addition to graphene, Ramer et al. reported that h-BN nanostructure arrays show polaritonic resonance with high-Q factors (up to 280) [154]. They investigated the field distribution associated with the polaritonic modes of h-BN nanostructures through AFM-IR. Figure 8(d) shows the field distributions of the bright and dark polaritonic modes of h-BN nanostructures. The dark modes are usually weak when detected by s-SNOM, indicating that AFM-IR is more suitable for probing polaritonic dark modes strongly confined within the nanostructure volume than s-SNOM. This is because AFM-IR has a high sensitivity to IR absorption inside the entire nanostructure, whereas s-SNOM exhibits surface sensitivity on the IR absorption of nanostructures. Unique polaritonic properties emerging in hyperbolic metasurfaces based on h-BN nanostructures have also been examined by Li et al., where concave wavefronts of polaritons in hyperbolic metasurfaces were successfully visualized [155]. Such polaritonic behavior has promising unique applications, including nanostructure-enhanced IR spectroscopy and planar hyperlenses.

## 7 Conclusion and outlook

After the global pandemic of coronavirus disease 2019 (COVID-19), demands for ultrasensitive biomolecular detection systems/devices have dramatically increased. For environmental concerns, detection technologies for small numbers of molecules in the surrounding environment have become crucial. SEIRA is one of the promising techniques to meet these demands, and particularly, resonant SEIRA is a promising technology to enhance the weak signals from a small number of molecules. These technologies are supported by the rapid development of nanofabrication techniques and have been drastically advanced in the past few decades. However, there are still several challenges for resonant SEIRA, which must be overcome before its commercialization. First, higher reproducibility of high and constant enhancement factors should be achieved for quantitative measurement. Scalable fabrication of MIR-active nanostructures using deep-ultraviolet lithography [156], laser interference lithography [157], and direct laser printing (writing) lithography [158, 159] is crucially important for practical implementation. Second, in the case of resonant SEIRA, the obtained IR absorption spectrum is affected by the plasmonic interaction between nanostructures and molecules, and it is different from the bulk spectrum, such as shifting the wavelength of absorption peaks or changing the ratio of the peak intensities. To understand these phenomena and fully utilize the potential of SEIRA, (i) theoretical analyses of the origin and detailed mechanism of spectroscopic properties of resonant SEIRA are crucial, (ii) the construction of an SEIRA spectra database is also important, (iii) the development of algorithms to recover material information from distorted spectra is necessary, and (iv) taking care of the vibrational mode spectral positions of analyte molecules and carefully tune the resonant frequency of the SEIRA structure are crucial (see Figure 1(a) of ref. [48] for characteristic infrared vibrations of various molecules). In summary, in this review, we considered only recent progress in IR spectroscopy and could not review other complementary vibrational spectroscopic techniques, such as surface-enhanced Raman spectroscopy (SERS) and tip-enhanced Raman spectroscopy (TERS). Raman spectroscopy provides complementary information of analytes; thus, it has been employed in several fields, such as the characterization of nanocarbon [160], [161], [162] and semiconductors [163, 164] and catalysis [165, 166]. Detailed working principles of SERS and TERS and their applications in the chemical characterization of nanostructured materials can be found in the literature [167], [168], [169].
